# Effects of Icodextrin Solution (Adept^®^) on Ovarian Cancer Cell Proliferation in an In Vitro Model

**DOI:** 10.3390/medicina58030386

**Published:** 2022-03-04

**Authors:** Wen-Hsin Chen, Hao Lin, Hung-Chun Fu, Chen-Hsuan Wu, Ching-Chou Tsai, Yu-Che Ou

**Affiliations:** 1 Department of Obstetrics and Gynecology, Chiayi Chang Gung Memorial Hospital and Chang Gung University College of Medicine, Chiayi 613, Taiwan; littlevicky7@gmail.com (W.-H.C.); allen133@cgmh.org.tw (H.-C.F.); 2 Department of Obstetrics and Gynecology, Kaohsiung Chang Gung Memorial Hospital and Chang Gung University College of Medicine, Kaohsiung 833, Taiwan; haolin@cgmh.org.tw (H.L.); chenhsuan5@gmail.com (C.-H.W.); nick58tsai@gmail.com (C.-C.T.)

**Keywords:** Adept^®^, anti-adhesion barrier, cell proliferation, cyclin, icodextrin, ovarian cancer

## Abstract

*Background and objective:* Anti-adhesion barriers are currently used during ovarian cancer surgery to decrease adhesion-related morbidity. Adept^®^ (4% icodextrin) solution, a liquid anti-adhesion material, has been widely used during gynecologic surgeries, though the risk of this barrier for oncologic surgery is controversial. The aim of this study was to determine the effect of Adept^®^ solution on the proliferation of ovarian cancer cells. *Materials and methods:* We assessed the dose- and time-dependent effects of icodextrin on the growth and proliferation of OVCAR-3 and A2780 human ovarian tumor cell lines in vitro. Cell growth was determined by cell number counting. Expressions of cell cycle-regulation proteins (cyclin D1 and cyclin B1) were determined using Western blot analysis. *Results:* Adept^®^ did not significantly increase ovarian cancer cell growth when tested at various concentrations (0, 1, 5, 10, 15, and 20%, equal to 0, 0.04, 0.2, 0.4, 0.6 and 0.8% icodextrin) and different time points (1–3 days) compared to control cells. Moreover, the protein levels of cyclin D1 and B1 were not overexpression-elevated in icodextrin-treated ovarian cancer cells, either with an increasing concentration or with an increasing treated time. These results demonstrated that Adept^®^ does not activate the growth or proliferation of ovarian cancer cells in either a dose- or time-dependent manner. *Conclusions:* This study supports the use of Adept^®^ solution as a safe anti-adhesion barrier for ovarian cancer surgery, though further in vivo studies are necessary.

## 1. Introduction

Complete cytoreductive surgery is the gold-standard treatment for ovarian cancer [1]. However, there is a risk that damage to the peritoneum and serosa after debulking surgery could lead to extensive intra-abdominal adhesions. These adhesions can lead to postoperative bowel obstructions and increase morbidity [2]. Moreover, adhesions can complicate second surgical procedures, which are frequently necessary in ovarian cancer due to their high recurrence rate [3,4]. Thus, the need to prevent intra-abdominal adhesions is an important issue. Previous reviews have described various strategies to reduce adhesions [5]. The use of anti-adhesion barriers is increasing, as their efficacy has been proven [6]. However, some adverse events related to anti-adhesive barriers have raised safety concerns, such as abscess formation, foreign body reactions, and increased risk of cancer progression [6]. For instance, the effect of a hyaluronic acid–carboxymethyl cellulose barrier (which was widely adopted in gynecologic fibroid surgery) on cancer cell proliferation has been questioned, as hyaluronic acid has been shown to be involved in tumor cell growth [7,8].

Adept^®^ (4% icodextrin solution) (Extraneal; Baxter Healthcare Inc., Utrecht, The Netherlands), an intra-abdominal anti-adhesive barrier instilled as a liquid agent, has proven effectiveness in adhesion prevention [9,10,11,12,13,14]. The mechanism of action is based on delayed intra-peritoneal absorption and a prolonged hydroflotation effect. The large glucose polymer component is degraded by alpha-amylase and absorbed through the lymphatic system into the systemic circulation. The alpha-amylase enzyme is present in human serum but not in the human peritoneum, which leads to slow absorption of Adept^®^ and a prolonged residual time of up to 3–5 days [12,15,16]. Moreover, the polymer creates a constant fluid interface between traumatized surfaces, which is thought to reduce initial adhesion formation at the critical early phase [12,17,18]. As icodextrin is a free-running liquid, a fluid reservoir easily forms in the whole abdominal cavity and covers the traumatized surfaces well. The utilization of icodextrin solution in benign gynecological surgery has been reported [5,10,11,12]. However, the application of icodextrin in ovarian cancer surgery is still controversial. Concerns exist about whether icodextrin promotes tumor growth, as it could potentially provide a medium in which cancer cells remaining after cytoreductive surgery could survive and proliferate, which could increase the risk of metastasis. There are few evaluations of the oncological risk of icodextrin solution in ovarian cancer.

Accordingly, we designed a preclinical study with in vitro experiments to analyze the effects of icodextrin solution on the proliferation of ovarian cancer cell lines. We aimed to provide an assessment of the appropriateness of icodextrin solution as an anti-adhesive agent in ovarian cancer.

## 2. Materials and Methods

### 2.1. Biomaterials

Adept^®^ (4% icodextrin) solution (Extraneal; Baxter Healthcare Inc., Utrecht, The Netherlands) was granted by the surgical department of Kaohsiung Chang Gung Memorial Hospital, without industrial funding.

### 2.2. Materials

Roswell Park Memorial Institute medium (RPMI 1640 medium), fetal bovine serum (FBS), trypsin/EDTA reagents, 100× antibiotic–antimycotic, and insulin (human recombinant, zinc solution, 4 mg/mL) were purchased from Invitrogen Corporation (Carlsbad, CA, USA). Acrylamide and protein molecular weight standard markers were obtained from Bio-Rad (Hercules, CA, USA). Radioimmunoprecipitation assay buffer (10×; RIPA lysis buffer) was purchased from Millipore Corporation (Billerica, MA, USA). The Enhanced Chemiluminescent (ECL) reagent kit was purchased from Thermo Scientific Inc. (Rockford, IL, USA). Anti-cyclin-B1 and cyclin D1/2 antibodies were obtained from Millipore Corporation (Billerica, MA, USA). Anti-β-actin antibody (AC-15) was purchased from Sigma Aldrich (St. Louis, MO, USA).

### 2.3. Cell Culture

The NIH ovarian adenocarcinoma cell lines OVCAR-3 and A2780 were obtained from the American Type Culture Collection (Rockville, MD, USA) and Bioresource Collection and Research Center (Hsinchu, Taiwan), respectively. OVCAR-3 cells were maintained in RPMI 1640 medium containing 20% FBS, 0.01 mg/mL recombinant human insulin and 1% antibiotic–antimycotic solution. A2780 cells were cultured in RPMI 1640 medium supplemented with 10% FBS and 1% antibiotic–antimycotic solution.

### 2.4. Cell Proliferation Assay

To examine the dose-dependent effect of Adept® solution on the cell growth of ovarian cancer cell lines, OVCAR-3 and A2780 cells were seeded at a density of 1.1 × 10^4^ cells/cm^2^ and 5.6 × 10^3^ cells/cm^2^, respectively, in triplicate, and cultured in regular medium for 3 days. Thereafter, cells were treated with either 1, 5, 10, 15 or 20% Adept^®^ (equal to 0.04, 0.2, 0.4, 0.6 or 0.8% icodextrin) solution in regular medium for 3 days. Meanwhile, control cells were treated with culture medium alone for 3 days. Live cell numbers were counted by the Trypan blue exclusion assay using a Cellometer Auto T4 Image-based cell counter (Nexcelom Bioscience, Lawrence, MA, USA). 

### 2.5. Determination of the Kinetics of Cell Growth

To examine the time-dependent effect of Adept^®^ solution on the growth of ovarian cancer cell lines, OVCAR-3 and A2780 cells were plated at a density of 1.1 × 10^4^ cells/cm^2^ and 4 × 10^3^ cells/cm^2^, respectively, in triplicate, and maintained in regular culture medium for 3 days. Then, the cells were cultured with regular medium alone (control cells) or treated with 10% Adept^®^ solution (equal to 0.4% icodextrin) in regular medium for 1, 2 or 3 days. Control or icodextrin-treated cells were harvested and counted after treatment on day 1 to 3. Similar results were obtained from at least 2 sets of independent experiments. At the endpoint of each harvest group, live cell numbers were counted by the Trypan blue exclusion assay using a Cellometer Auto T4 Image-based cell counter (Nexcelom Bioscience, Lawrence, MA, USA).

### 2.6. Immunoblotting 

For both the dose- and time-dependent manners, except the cell number counting, immunoblotting was applied at each endpoint. To detect cellular proteins, cells were harvested, counted, and lysed with RIPA lysis buffer containing protease and phosphatase inhibitors. Aliquots of total cellular lysates (20–60 μg protein) were subjected to electrophoresis on SDS-polyacrylamide gels (7.5–12% acrylamide) and then transferred to nitrocellulose membranes for Western blot analyses. The membrane filters were blocked in 5% skimmed milk for 1 h at room temperature and subsequently incubated with corresponding primary and secondary antibodies, including anti-cyclin D1 monoclonal antibody (1:1000, 3 h, room temperature) and anti-cyclin B1 monoclonal antibody (1:1000, 3 h, room temperature). The proteins of interest were visualized by an ECL detection system, and β-actin (1:20,000, 1 h, room temperature) was used as loading control.

### 2.7. Statistical Analysis

Each set of experiments was performed in triplicate, as specified in the figure legend or experimental design, and repeated at least two or three times as independent experiments. The mean and standard deviation (SD) were calculated. The Student’s t-test was used for comparisons between the treated and control groups. For dose-dependent manner, each different dose-treated group was compared with the control group. For time-dependent manner, the treated group was compared to the control group with the same culture time. For this entire study, *p*-values lower than 0.05 were considered statistically significant.

## 3. Results

### 3.1. Adept^®^ Solution Does Not Affect Ovarian Cancer Cell Growth in Dosage and Kinetic Manner

OVCAR-3 and A2780 ovarian cancer cell lines were treated with Adept^®^ solution at different ratios (*v*/*v*; 0, 1, 5, 10, 15, and 20%, equal to 0, 0.04, 0.2, 0.4, 0.6 and 0.8% icodextrin) and treated with 10% Adept^®^ solution (*v*/*v*, equal to 0.4% icodextrin) for various periods of time (1, 2, and 3 days).

**Figure 1:**
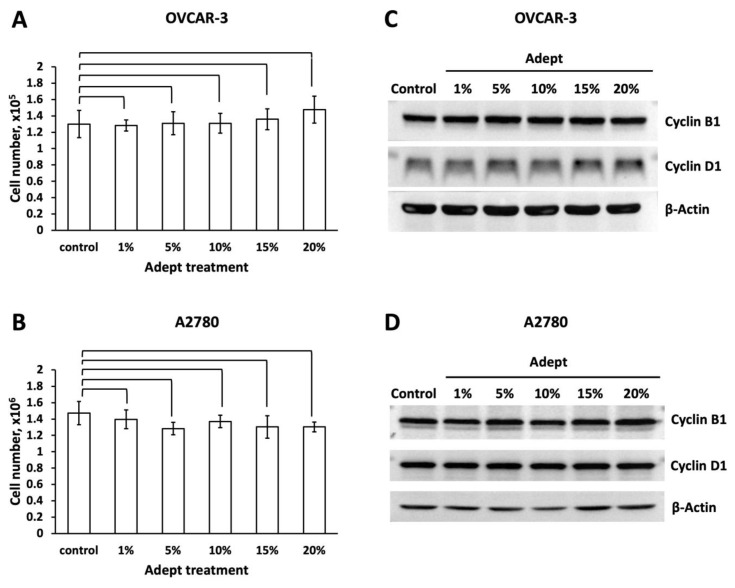
**Various concentrations of Adept^®^ solution do not affect OVCAR-3 and A2780 cell growth and proliferation.** (**A**) OVCAR-3 and (**B**) A2780 cells were plated in six-well plates at 1.1 × 10^4^ cells/cm^2^ and 5.6 × 10^3^ cells/cm^2^, respectively, in regular medium for 3 days, then incubated in 1, 5, 10, 15 or 20% Adept^®^ (equal to 0, 0.04, 0.2, 0.4, 0.6 and 0.8% icodextrin) solution for 3 days; control cells were cultured in medium alone. After treatment for 3 days, cells were trypsinized and total live cell numbers were counted. The ratio of cell growth was calculated by normalizing the numbers of treated cells to the number of control cells (*n* = 3 × 2). Total cell lysates from (**C**) OVCAR-3 and (**D**) A2780 cells treated with various concentrations of Adept^®^ solution were subjected to Western blot analysis to quantify the cell cycle regulation proteins cyclin B1 and cyclin D1; β-actin was used as a loading control.

For the dose-dependent experiment, cells were treated with 0–20% Adept^®^ solution for 3 days, and cell growth was analyzed. As the results showed (Table 1, Figure 1A,B), both cancer cell lines survived in different ratios of Adept^®^ solution. Compared with the control cells (RPMI medium alone), no significant difference in OVCAR-3 or A2780 cell numbers was observed in either ratio of icodextrin-treated medium. 

**Figure 2:**
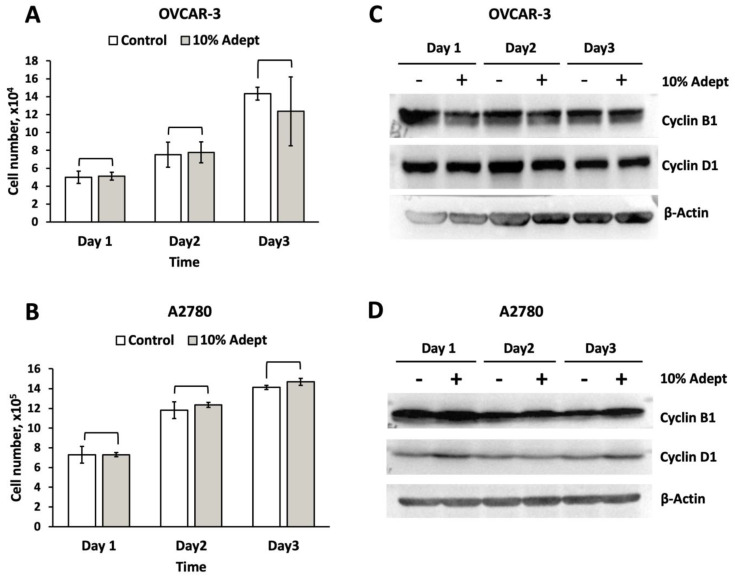
**Adept^®^ solution does not affect OVCAR-3 and A2780 cell growth and proliferation at various time-points.** (**A**) OVCAR-3 and (**B**) A2780 cells were plated in six-well plates in triplicate at 1.1 × 10^4^ cells/cm^2^ and 4 × 10^3^ cells/cm^2^, respectively, in regular medium for 3 days, then 10% Adept^®^ solution (*v*/*v*, equal to 0.4% icodextrin) was added; control cells were cultured in regular medium alone. Triplicate samples were harvested after 1, 2 or 3 days of icodextrin treatment, and then cells were trypsinized. Total live cell numbers were counted. The ratio of cell growth was calculated by normalizing the numbers of treated cells to the number of control cells (*n* = 3 × 2). Total cell lysates from (**C**) OVCAR-3 and (**D**) A2780 cells at various time points were subjected to Western blot analysis to quantify the cell cycle regulation proteins cyclin B1 and cyclin D1; β-actin was used as a loading control.

Moreover, for the time-dependent manner, as shown in Table 2, Figure 2A,B, we observed no significant difference in the numbers of OVCAR-3 and A2780 cells treated with 10% Adept^®^ solution (*v*/*v*, equal to 0.4% icodextrin) compared to the control cells at various time points (1–3 days).

### 3.2. Adept^®^ Solution Does Not Upregulate Protein Markers of Ovarian Tumor Cell Proliferation

To further investigate the effect of Adept^®^ solution on OVCAR-3 and A2780 cell proliferation, Western blot analysis was conducted to analyze the levels of the cell cycle regulation marker proteins cyclin B1 and cyclin D1. Neither of these proteins were significantly affected by any concentration of Adept^®^ solution (1, 5, 10, 15, and 20%, equal to 0.04, 0.2, 0.4, 0.6 and 0.8% icodextrin) tested in either OVCAR-3 or A2780 cells (Figure 1C,D). Likewise, icodextrin did not significantly alter the expression of cyclin B1 or cyclin D1 in a time-dependent manner in either cancer cell line (Figure 2C,D).

## 4. Discussion

This study shows that Adept^®^ solution does not induce the proliferation of OVCAR-3 or A2780 ovarian tumor cell lines in vitro. When clinically peri-operatively administered, the free-running liquid icodextrin can easily form a fluid reservoir throughout the entire peritoneal cavity. Icodextrin is metabolized by amylase, which is not present in the human peritoneal cavity; thus, this barrier has a prolonged residence time of up to 3–5 days [15,16]. Moreover, free-floating cancer cells may be suspended and stay alive in the icodextrin solution. In order to model the clinical exposure, we investigated the effects of various concentrations of icodextrin solution on ovarian cancer cell proliferation over 3 days. However, it is difficult to translate the in vitro concentration to reach the working concentration in human body. The osmotic changes of the retained intra-abdominal icodextrin solution could be varied dynamically during the absorption [19], since the simultaneously existed fluid in the peritoneal cavity was hard to be estimated. In particular, peritoneal lavages during surgery and the development of ascites could cause a decreasing concentration of Adept^®^ solution. Thus, the stepwise dilutions of Adept^®^ solution were used. In the cell culture model, we found ovarian cancer cells cannot survive in 100% Adept^®^ solution, without FBS or culture medium. Furthermore, we found the culture medium with higher than 20% Adept^®^ inhibited the survival of ovarian cancer cells. When treated with decreasing concentrations of Adept^®^ solution (1, 5, 10, 15 or 20%), our data revealed that the ovarian cancer cells survived in icodextrin solution, but that icodextrin did not activate the growth and proliferation of the cell lines in either a dose- or time-dependent manner. 

Ovarian cancer actually comprises heterogenous histotypes with distinct cellular origin, tumor behavior and different patterns of disease progression between patients [20]. Hence, it was necessary to employ the cell lines to represent this variability. High-grade serous carcinoma is one of the most common subtypes of epithelial ovarian carcinoma. These tumors typically present at an advanced stage, are fast growing, and easily spread throughout the peritoneal cavity. Non-serous histologic ovarian cancer, including endometrioid, clear cell and mucinous carcinoma, generally present as early-stage disease, but also exhibit invasive behavior and do not respond well to traditional ovarian chemotherapeutic agents [21]. We used the OVCAR-3 and A2780 cell lines in the present study to represent this variability. OVCAR3 cells are derived from the ascites of a high-grade serous tumor, and are typically clinically aggressive, able to survive and grow in suspension, and have a high tumorigenic potential [21,22]. A2780 cells are derived from an untreated early-stage primary non-serous tumor before metastasis has occurred. A2780 cells can aggressively invade collagen, but not Matrigel [21]. These discrepancies between the two cancer cell lines represent the varied histologic types and aggressiveness observed in ovarian carcinoma. Similarly, another study [8] that evaluated the effects of an anti-adhesive barrier on ovarian tumor progression used a variety of cell lines from high-grade serous tumors and non-serous tumors, which supports the importance of distinguishing the variability of ovarian carcinoma. Overall, our results indicate that icodextrin does not modulate the cell proliferation of either cancer cell line tested, which suggests that icodextrin does not promote the proliferation of different subtypes of ovarian cancer with distinct behavioral characteristics. 

We also analyzed two cell cycle regulation marker proteins, cyclin B1 and cyclin D1. Cyclin-B1 and cyclin-D1 have been published as crucial regulators of cell proliferation [23,24]. Altered regulation of the cell cycle results in uncontrolled cell proliferation, which is the hallmark of cancer generation. Cell cycle progression is governed by a series of cyclins, which bind the cyclin-dependent kinases (CDK) and drive the cell cycle [23,24]. Cyclin D1 promotes the G1 to the S phase transformation of the cell cycle by forming a complex with CDK4. Furthermore, cyclin D1 binds to and activates CDK4/CDK6 to phosphorylate the protein Rb, which promotes the transition of the cell cycle to the S phase [24]. On the other hand, cyclin B1 is important in regulating the G2-to-M phase transition, which is essential for cellular mitosis, and altered expression of cyclin B1 is implicated in the aggressive proliferation of cancer cells [24]. A previous in vitro study showed that overexpression of mortalin (a stress response-related glucose-regulated protein) mediated ovarian cancer cell (A2780) proliferation by inducing abnormal expression of cyclin-B1 and cyclin-D1 [25]. Thus, the evidence indicates that the frequent amplification and overexpression of cyclin D1 and B1 in cancer cells correlate with rapid cell proliferation. Therefore, in the present study, the lack of significant overexpression of the cyclin D1 or B1 proteins in the icodextrin-treated ovarian cancer cell lines, in either a dose- or time-dependent manner, further strongly suggests that icodextrin does not promote ovarian cancer cell proliferation. 

Similarly to this work, previous studies on colon cancer demonstrated that icodextrin did not activate the proliferation of cancer cells (Table 3). An in vivo study by Van den Tol et al. [26], revealed intra-abdominal instillation of 7.5% icodextrin did not increase total peritoneal tumor load at 21 days compared with the no-instillation groups in a rat model of colon carcinoma. Jouvin et al. [27] demonstrated no significant difference in the cell migration rate of 50% icodextrin-treated colon cancer cells and control cells in vitro. Furthermore, in a murine model of induced peritoneal carcinomatosis, the peritoneal carcinomatosis index score was not significantly different between the intra-abdominal 4% icodextrin instillation and no-instillation groups after 15 days. Additionally, some studies have even reported that icodextrin inhibits cancer cell proliferation. An in vitro growth assay established by Van den Tol et al. [26] even showed a significant reduction in growth of the tumor cells with 4% icodextrin solution. Al Dybiat et al. [28] revealed that, in a mouse model of peritoneally injected colon cancer, the bioluminescent signals of cancer cells were significantly lower in the 4% icodextrin-treated group than the control group at 7 and 14 days. Moreover, 4% icodextrin treatment significantly decreased the peritoneal carcinomatosis index score at 14 days compared with the non-treated group. In the current study, we did not observe that icodextrin inhibited ovarian cancer cell proliferation. This discrepancy may possibly be due to the heterogeneity of colon and ovarian cancer cells and the different study protocols and durations. Overall, we believe that our results obtained using ovarian cancer cells are encouraging, and support further evaluation of icodextrin as an anti-adhesion barrier for ovarian cancer surgeries.

The strength of the present study was that the icodextrin solution we adopted was diluted from the original Adept^®^ solution from the manufacturer. The experiment would be more representative for the clinical application. There are several limitations to our study. First, in vivo experiments are lacking. Alpha-amylase, which metabolizes icodextrin to oligosaccharide molecules for absorption, is present in the peritoneum of mice and rabbits, but not in the human peritoneum. The biocompatibility of icodextrin may vary between animal models and human patients. Thus, the clinical effects of icodextrin on ovarian cancer warrant further investigation. Second, we established that icodextrin appeared not to promote ovarian cancer cell proliferation by assessment of the expression of cyclin D1 and B1 proteins, the two crucial cell proliferation markers. However, more detailed assessments of cell cycle regulation could be further investigated, such as the measurements of the DNA contents of cells and cell numbers in the various cell cycle phases. Third, the time of incubation of cells might be extended to assess more complete cell growth changes, considering that the Adept^®^ exposure time could be varied under differing absorption capacities of the peritoneum in different individuals.

## 5. Conclusions

The anti-adhesion agent Adept^®^ solution did not significantly increase the proliferation of ovarian cancer cells in vitro. These results indicate that Adept^®^ solution could be adopted postoperatively during ovarian cancer cytoreductive surgery as a neutral substance to prevent adhesions, without promoting or inhibiting ovarian cancer cell progression. Although not evaluated in this study, the impact of Adept^®^ solution on the survival outcomes of patients with ovarian malignancies could be investigated in the future.

## Figures and Tables

**Table 1:** Dose-dependent effect of Adept^®^ on the cell growth of OVCAR-3 and A2780 cell.

	OVCAR-3 Cell (×10^5^)	*p*-Value	A2780 Cell (×10^6^)	*p*-Value
Control	1.30 ± 0.17		1.47 ± 0.14	
1% Adept^®^ (0.04% icodextrin)	1.28 ± 0.07	0.88	1.40 ± 0.11	0.51
5% Adept^®^ (0.2% icodextrin)	1.31 ± 0.14	0.94	1.28 ± 0.08	0.11
10% Adept^®^ (0.4% icodextrin)	1.31 ± 0.12	0.94	1.37 ± 0.08	0.33
15% Adept^®^ (0.6% icodextrin)	1.36 ± 0.13	0.65	1.30 ± 0.14	0.21
20% Adept^®^ (0.8% icodextrin)	1.48 ± 0.16	0.26	1.30 ± 0.06	0.13

Data are reported as the mean ± SD for experiments performed in triplicate. OVCAR-3 and A2780 cells were treated with different doses of Adept^®^ solution, 1, 5, 10, 15 or 20% Adept^®^ (equal to 0.04, 0.2, 0.4, 0.6 or 0.8% icodextrin) and analyzed after 3 days. * *p* < 0.05 for Adept^®^ treated cells vs. control (Student’s *t*-test).

**Table 2:** Time-dependent effect of 10% Adept^®^ on the cell growth of OVCAR-3 and A2780 cell.

	OVCAR-3 Cell (×10^4^)			A2780 Cell (×10^5^)		
	Control	10% Adept^®^ (0.4% Icodextrin)	*p*-Value	Control	10% Adept^®^ (0.4% Icodextrin)	*p*-Value
1 day	4.99 ± 0.69	5.12 ± 0.43	0.80	7.29 ± 0.85	7.31 ± 0.22	0.98
2 day	7.52 ± 1.40	7.78 ± 1.16	0.82	11.83 ± 0.85	12.36 ± 0.25	0.35
3 day	14.33 ± 0.71	12.36 ± 3.84	0.43	14.13 ± 0.21	14.70 ± 0.35	0.07

Data are reported as the mean ± SD for experiments performed in triplicate. OVCAR-3 and A2780 cells were treated with 10% Adept^®^ (equal 0.4% icodextrin) and cultured for 1, 2 and 3 days. The data were analyzed at each endpoint.

**Table 3:** Summary of published studies of the effectiveness of icodextrin solution on cancer cell progression.

Study	Cancer Type	In Vitro/In Vivo	Target/Control	Outcome Measure	Summary of Outcome
van den Tol et al. (2005) [26]	Colon cancer	In vitro CC531 tumor cell	1%, 2%, 4% icodextrin/RPMI	Tumor cell DNA at 2, 4, and 6 days	(1) 4% Icodextrin reduced tumor growth after 4 and 6 days versus control group. (*p* < 0.001) (2) 2% and 1% Icodextrin had no difference on tumor growth versus control group. (*p* > 0.05)
		In vivo murine model with peritoneal trauma + CC531 cell	7.5% icodextrin/RPMI/no instillation	PCI score at 21 days	(1) No difference in total score in 3 groups (*p* > 0.05) (2) 7.5% icodextrin has lower tumor score at the retroperitoneum site versus control group (*p* = 0.007)
Jouvin et al. (2017) [27]	Colon cancer	In vitro CT26 LUC cell	30%, 50%, 70%, 90% icodextrin/RPMI	Tumor cell viability and growth at 1, 2, and 3 days	Viability significantly lower at day 2 and 3 in icodextrin group versus control group (*p* < 0.001)
			50% icodextrin/RPMI	Cell migration rate at 4, 6, 8, 10 and 24 h	No difference between groups at the different times (*p* > 0.05)
		In vivo murine model + CT26 LUC cell	4% icodextrin/no instillation	PCI score at 15 days	No difference in PCI score between icodextrin and control group (*p* = 0.2)
Al Dybiat et al. (2020) [28]	Colon cancer	In vivo murine model + CT26 LUC cell	4% icodextrin/no instillation	PCI score and bioluminescence signal of tumor at 14–21 days	Significant decrease after icodextrin treatment (*p* < 0.017)
Chen et al. (current study)	Ovarian cancer	In vitro A2780 cell OVCAR-3 cell	1, 5, 10, 15, 20% Adept^®^ (=0.04, 0.2, 0.4, 0.6, 0.8% icodextrin)/no instillation	Tumor cell growth and immunoblotting at 3 days	No significant difference between each dose-treated group and control group (*p* > 0.05)
			10% Adept^®^ (=0.4% icodextrin)/no instillation	Tumor cell growth and immunoblotting at 1, 2, and 3 days	No significant difference between the treated group and control group at each endpoint (*p* > 0.05)
